# (2*E*)-2-[(2*H*-1,3-Benzodioxol-5-yl)methyl­idene]-2,3-dihydro-1*H*-inden-1-one

**DOI:** 10.1107/S1600536812009464

**Published:** 2012-03-10

**Authors:** Abdullah M. Asiri, Hassan M. Faidallah, Khulud F. Al-Nemari, Seik Weng Ng, Edward R. T. Tiekink

**Affiliations:** a Chemistry Department, Faculty of Science, King Abdulaziz University, PO Box 80203, Jeddah, Saudi Arabia; b The Center of Excellence for Advanced Materials Research, King Abdulaziz University, Jeddah, PO Box 80203, Saudi Arabia; c Department of Chemistry, University of Malaya, 50603 Kuala Lumpur, Malaysia

## Abstract

In the title compound, C_17_H_12_O_3_, each of the five-membered rings in the inden-1-one and 1,3-benzodioxole residues is almost planar (r.m.s. deviations = 0.041 and 0.033 Å, respectively). A small twist about the single bond linking the two residues is evident [the C—C—C—C torsion angle = 8.7 (4)°]. Supra­molecular zigzag layers propagating in the *ac* plane are formed in the crystal *via* C—H⋯O inter­actions. The layers are linked *via* π–π inter­actions between the five- and six-membered rings of 1,3-benzodioxole residues [centroid–centroid distance = 3.4977 (14) Å].

## Related literature

For the biological activity of related species, see: Vera-DiVaio *et al.* (2009 ▶). For related structures, see: Asiri *et al.* (2012*a* ▶,*b* ▶).
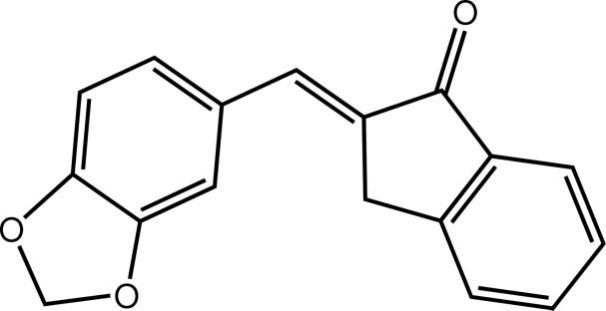


## Experimental

### 

#### Crystal data

C_17_H_12_O_3_
*M* *_r_* = 264.27
Orthorhombic, 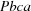

*a* = 12.6102 (12) Å
*b* = 7.3497 (10) Å
*c* = 26.569 (4) Å
*V* = 2462.5 (5) Å^3^
*Z* = 8
Mo *K*α radiation
μ = 0.10 mm^−1^
*T* = 100 K
0.35 × 0.10 × 0.05 mm

#### Data collection

Agilent SuperNova Dual diffractometer with an Atlas detector
Absorption correction: multi-scan (*CrysAlis PRO*; Agilent, 2011 ▶) *T*_min_ = 0.967, *T*_max_ = 0.995
6424 measured reflections
2820 independent reflections
1697 reflections with *I* > 2σ(*I*)
*R*_int_ = 0.057

#### Refinement

*R*[*F* ^2^ > 2σ(*F* ^2^)] = 0.055
*wR*(*F* ^2^) = 0.136
*S* = 0.98
2820 reflections
181 parameters
H-atom parameters constrained
Δρ_max_ = 0.26 e Å^−3^
Δρ_min_ = −0.26 e Å^−3^

### 

Data collection: *CrysAlis PRO* (Agilent, 2011 ▶); cell refinement: *CrysAlis PRO*; data reduction: *CrysAlis PRO*; program(s) used to solve structure: *SHELXS97* (Sheldrick, 2008 ▶); program(s) used to refine structure: *SHELXL97* (Sheldrick, 2008 ▶); molecular graphics: *ORTEP-3* (Farrugia, 1997 ▶) and *DIAMOND* (Brandenburg, 2006 ▶); software used to prepare material for publication: *publCIF* (Westrip, 2010 ▶).

## Supplementary Material

Crystal structure: contains datablock(s) global, I. DOI: 10.1107/S1600536812009464/hb6667sup1.cif

Structure factors: contains datablock(s) I. DOI: 10.1107/S1600536812009464/hb6667Isup2.hkl

Supplementary material file. DOI: 10.1107/S1600536812009464/hb6667Isup3.cml

Additional supplementary materials:  crystallographic information; 3D view; checkCIF report

## Figures and Tables

**Table 1:** Hydrogen-bond geometry (Å, °)

*D*—H⋯*A*	*D*—H	H⋯*A*	*D*⋯*A*	*D*—H⋯*A*
C5—H5⋯O1^i^	0.95	2.47	3.290 (3)	144
C17—H17*A*⋯O1^ii^	0.99	2.46	3.302 (3)	143

Symmetry codes: (i) 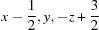; (ii) 
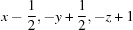
.

**Fig. 1.**
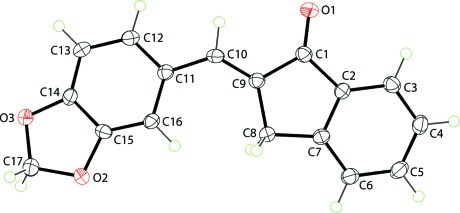
The molecular structure of (I) showing displacement ellipsoids at the 50% probability level.

**Fig. 2.**
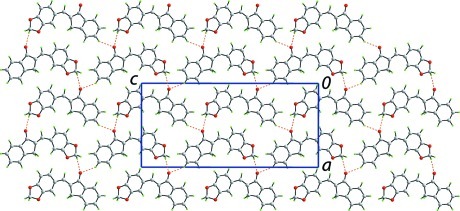
A view of the supramolecular layer in (I) in the ac plane. The C—H···O interactions are shown as orange dashed lines.

**Fig. 3.**
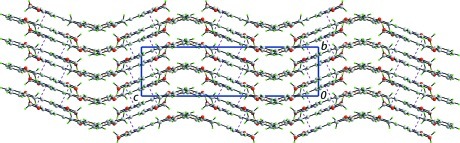
A view in projection down the a axis of the unit-cell contents of (I) showing the stacking of zigzag layers. The C—H···O and π–π interactions are shown as orange and purple dashed lines, respectively.

## supplementary crystallographic information

### Comment

The crystal and molecular structure of the title compound, 2-benzo[1,3]dioxol-5-ylmethylene-indan-1-one (I), has been determined in connection with recent structural studies on related derivatives (Asiri *et al.*, 2012*a*; Asiri *et al.*, 2012*b*). The motivation for the original synthesis was its relationship to biologically active compounds (Vera-DiVaio *et al.*, 2009).

In the molecule of (I), Fig. 1, both five-membered rings are essentially planar. In the inden-1-one residue the r.m.s. deviation for the five atoms = 0.041 Å [maximum deviations = 0.033 (2) for the C8 atom and -0.033 (2) for the C7 atom] and in the 1,3-benzodioxole residue, the r.m.s. deviation = 0.033 Å [maximum deviations = 0.028 (3) [C17] and -0.028 (1) [O3]]. A twist in the molecule about the C10—C11 bond is evident with the C9—C10—C11—C16 torsion angle being 8.7 (4)°. The configuration about the C9═C10 bond [1.340 (3) Å] is *E*.

In the crystal packing, C—H···O interactions, Table 1, involving the bifurcated carbonyl-O atom link molecules into zigzag layers in the *ac* plane, Fig. 2. Layers are linked along the *b* axis *via*π–π interactions between the five- and six-membered rings of 1,3-benzodioxole residues [ring centroid···centroid distance = 3.4977 (14) Å, angle of inclination = 10.97 (12)° for symmetry operation 3/2 - *x*, -1/2 + *y*, *z*], Fig. 3.

### Experimental

A solution of the piperonaldehyde (1.5 g, 0.01 *M*) in ethanol (20 ml) was added to a stirred solution of 1-indanone (1.3 g, 0.01 *M*) in (20%) ethanolic KOH (20 ml), and stirring was maintained at room temperature for 6 h. The reaction mixture was then poured into water (200 ml) and set aside overnight. The precipitated solid product was collected by filtration, washed with water, dried and recrystallized from ethanol as light-yellow prisms. Yield: 93%; *M*. pt: 450–451 K.

### Refinement

Carbon-bound H-atoms were placed in calculated positions [C—H = 0.95 to 0.99 Å, *U*_iso_(H) = 1.2*U*_eq_(C)] and were included in the refinement in the riding model approximation.

### Crystal data

**Table:**

C_17_H_12_O_3_	*F*(000) = 1104
*M**_r_* = 264.27	*D*_x_ = 1.426 Mg m^−^^3^
Orthorhombic, *P**b**c**a*	Mo *K*α radiation, λ = 0.71073 Å
Hall symbol: -P 2ac 2ab	Cell parameters from 930 reflections
*a* = 12.6102 (12) Å	θ = 2.8–27.5°
*b* = 7.3497 (10) Å	µ = 0.10 mm^−^^1^
*c* = 26.569 (4) Å	*T* = 100 K
*V* = 2462.5 (5) Å^3^	Prism, light-yellow
*Z* = 8	0.35 × 0.10 × 0.05 mm

### Data collection

**Table:**

Agilent SuperNova Dual diffractometer with an Atlas detector	2820 independent reflections
Radiation source: SuperNova (Mo) X-ray Source	1697 reflections with *I* > 2σ(*I*)
Mirror monochromator	*R*_int_ = 0.057
Detector resolution: 10.4041 pixels mm^-1^	θ_max_ = 27.6°, θ_min_ = 3.2°
ω scan	*h* = −16→8
Absorption correction: multi-scan (*CrysAlis PRO*; Agilent, 2011)	*k* = −9→6
*T*_min_ = 0.967, *T*_max_ = 0.995	*l* = −34→22
6424 measured reflections	

### Refinement

**Table:**

Refinement on *F*^2^	Primary atom site location: structure-invariant direct methods
Least-squares matrix: full	Secondary atom site location: difference Fourier map
*R*[*F*^2^ > 2σ(*F*^2^)] = 0.055	Hydrogen site location: inferred from neighbouring sites
*wR*(*F*^2^) = 0.136	H-atom parameters constrained
*S* = 0.98	*w* = 1/[σ^2^(*F*_o_^2^) + (0.0502*P*)^2^] where *P* = (*F*_o_^2^ + 2*F*_c_^2^)/3
2820 reflections	(Δ/σ)_max_ = 0.001
181 parameters	Δρ_max_ = 0.26 e Å^−^^3^
0 restraints	Δρ_min_ = −0.26 e Å^−^^3^

### Fractional atomic coordinates and isotropic or equivalent isotropic displacement parameters (Å^2^)

**Table:**

	*x*	*y*	*z*	*U*_iso_*/*U*_eq_	
O1	0.92949 (12)	0.5693 (2)	0.65488 (6)	0.0275 (4)	
O2	0.61242 (12)	0.2489 (2)	0.41852 (5)	0.0283 (4)	
O3	0.75515 (12)	0.1445 (2)	0.37080 (6)	0.0283 (4)	
C1	0.83441 (19)	0.5422 (3)	0.64704 (8)	0.0212 (5)	
C2	0.74657 (18)	0.5690 (3)	0.68310 (8)	0.0197 (5)	
C3	0.75035 (19)	0.6274 (3)	0.73287 (8)	0.0231 (5)	
H3	0.8154	0.6647	0.7476	0.028*	
C4	0.65715 (19)	0.6298 (3)	0.76036 (8)	0.0267 (6)	
H4	0.6578	0.6709	0.7943	0.032*	
C5	0.5625 (2)	0.5723 (3)	0.73861 (9)	0.0291 (6)	
H5	0.4996	0.5711	0.7583	0.035*	
C6	0.55825 (19)	0.5165 (3)	0.68870 (8)	0.0263 (6)	
H6	0.4931	0.4781	0.6742	0.032*	
C7	0.65094 (18)	0.5179 (3)	0.66035 (8)	0.0210 (5)	
C8	0.66690 (17)	0.4682 (3)	0.60554 (8)	0.0200 (5)	
H8A	0.6391	0.3449	0.5983	0.024*	
H8B	0.6314	0.5569	0.5831	0.024*	
C9	0.78635 (18)	0.4750 (3)	0.59906 (8)	0.0199 (5)	
C10	0.84909 (18)	0.4281 (3)	0.56046 (8)	0.0207 (5)	
H10	0.9227	0.4445	0.5665	0.025*	
C11	0.82372 (18)	0.3562 (3)	0.51066 (8)	0.0194 (5)	
C12	0.90839 (19)	0.2975 (3)	0.48054 (8)	0.0254 (5)	
H12	0.9785	0.3069	0.4934	0.030*	
C13	0.89343 (19)	0.2255 (3)	0.43228 (8)	0.0266 (5)	
H13	0.9514	0.1870	0.4121	0.032*	
C14	0.79071 (19)	0.2135 (3)	0.41575 (8)	0.0221 (5)	
C15	0.70630 (17)	0.2729 (3)	0.44464 (8)	0.0197 (5)	
C16	0.71892 (18)	0.3447 (3)	0.49167 (8)	0.0210 (5)	
H16	0.6599	0.3851	0.5109	0.025*	
C17	0.64264 (19)	0.1752 (4)	0.37035 (8)	0.0296 (6)	
H17A	0.6048	0.0593	0.3642	0.036*	
H17B	0.6239	0.2616	0.3432	0.036*	

### Atomic displacement parameters (Å^2^)

**Table:**

	*U*^11^	*U*^22^	*U*^33^	*U*^12^	*U*^13^	*U*^23^
O1	0.0173 (9)	0.0395 (10)	0.0256 (9)	−0.0010 (7)	−0.0025 (7)	−0.0025 (8)
O2	0.0210 (9)	0.0432 (11)	0.0206 (8)	−0.0023 (8)	−0.0017 (7)	−0.0073 (7)
O3	0.0210 (9)	0.0422 (10)	0.0217 (8)	−0.0029 (8)	0.0018 (7)	−0.0092 (8)
C1	0.0204 (13)	0.0226 (12)	0.0206 (11)	0.0005 (10)	−0.0012 (10)	0.0010 (9)
C2	0.0202 (12)	0.0193 (11)	0.0196 (11)	0.0004 (10)	0.0017 (10)	0.0007 (9)
C3	0.0221 (13)	0.0265 (12)	0.0207 (11)	−0.0010 (10)	−0.0025 (10)	0.0012 (10)
C4	0.0299 (14)	0.0312 (13)	0.0189 (11)	0.0050 (11)	0.0016 (11)	−0.0016 (10)
C5	0.0233 (14)	0.0379 (15)	0.0261 (12)	0.0057 (11)	0.0056 (11)	0.0000 (11)
C6	0.0205 (13)	0.0352 (14)	0.0231 (12)	0.0021 (11)	0.0006 (10)	0.0018 (10)
C7	0.0195 (12)	0.0203 (11)	0.0230 (11)	0.0021 (10)	0.0002 (10)	0.0009 (9)
C8	0.0183 (12)	0.0218 (11)	0.0200 (11)	−0.0009 (10)	0.0008 (10)	−0.0004 (9)
C9	0.0186 (12)	0.0214 (11)	0.0199 (11)	0.0002 (10)	−0.0001 (10)	0.0015 (9)
C10	0.0156 (12)	0.0231 (12)	0.0234 (11)	−0.0021 (9)	−0.0010 (10)	0.0030 (10)
C11	0.0195 (12)	0.0195 (11)	0.0191 (10)	−0.0012 (10)	0.0013 (10)	0.0026 (9)
C12	0.0177 (13)	0.0333 (13)	0.0251 (11)	−0.0028 (10)	0.0009 (10)	0.0006 (10)
C13	0.0183 (13)	0.0353 (13)	0.0260 (12)	−0.0006 (11)	0.0052 (11)	−0.0019 (10)
C14	0.0231 (13)	0.0257 (12)	0.0175 (10)	−0.0013 (10)	0.0038 (10)	−0.0005 (10)
C15	0.0169 (12)	0.0209 (11)	0.0213 (11)	−0.0027 (9)	−0.0028 (10)	0.0026 (9)
C16	0.0183 (12)	0.0252 (12)	0.0196 (11)	−0.0017 (10)	0.0029 (10)	0.0024 (10)
C17	0.0248 (14)	0.0421 (16)	0.0218 (11)	0.0053 (11)	−0.0002 (11)	−0.0046 (11)

### Geometric parameters (Å, º)

**Table:**

O1—C1	1.233 (3)	C8—C9	1.517 (3)
O2—C15	1.384 (3)	C8—H8A	0.9900
O2—C17	1.441 (3)	C8—H8B	0.9900
O3—C14	1.373 (3)	C9—C10	1.340 (3)
O3—C17	1.437 (3)	C10—C11	1.460 (3)
C1—C2	1.478 (3)	C10—H10	0.9500
C1—C9	1.496 (3)	C11—C12	1.402 (3)
C2—C3	1.391 (3)	C11—C16	1.417 (3)
C2—C7	1.400 (3)	C12—C13	1.400 (3)
C3—C4	1.384 (3)	C12—H12	0.9500
C3—H3	0.9500	C13—C14	1.371 (3)
C4—C5	1.392 (3)	C13—H13	0.9500
C4—H4	0.9500	C14—C15	1.383 (3)
C5—C6	1.389 (3)	C15—C16	1.366 (3)
C5—H5	0.9500	C16—H16	0.9500
C6—C7	1.391 (3)	C17—H17A	0.9900
C6—H6	0.9500	C17—H17B	0.9900
C7—C8	1.515 (3)		
			
C15—O2—C17	105.49 (17)	C10—C9—C8	131.7 (2)
C14—O3—C17	105.78 (16)	C1—C9—C8	108.44 (18)
O1—C1—C2	126.7 (2)	C9—C10—C11	131.1 (2)
O1—C1—C9	126.3 (2)	C9—C10—H10	114.5
C2—C1—C9	107.04 (19)	C11—C10—H10	114.5
C3—C2—C7	121.5 (2)	C12—C11—C16	119.2 (2)
C3—C2—C1	129.2 (2)	C12—C11—C10	117.5 (2)
C7—C2—C1	109.26 (19)	C16—C11—C10	123.3 (2)
C4—C3—C2	118.5 (2)	C13—C12—C11	122.4 (2)
C4—C3—H3	120.8	C13—C12—H12	118.8
C2—C3—H3	120.8	C11—C12—H12	118.8
C3—C4—C5	120.3 (2)	C14—C13—C12	116.4 (2)
C3—C4—H4	119.8	C14—C13—H13	121.8
C5—C4—H4	119.8	C12—C13—H13	121.8
C6—C5—C4	121.3 (2)	C13—C14—O3	127.7 (2)
C6—C5—H5	119.4	C13—C14—C15	121.9 (2)
C4—C5—H5	119.4	O3—C14—C15	110.4 (2)
C7—C6—C5	118.9 (2)	C16—C15—C14	122.7 (2)
C7—C6—H6	120.6	C16—C15—O2	127.4 (2)
C5—C6—H6	120.6	C14—C15—O2	109.86 (19)
C6—C7—C2	119.5 (2)	C15—C16—C11	117.2 (2)
C6—C7—C8	129.1 (2)	C15—C16—H16	121.4
C2—C7—C8	111.42 (19)	C11—C16—H16	121.4
C9—C8—C7	103.49 (17)	O3—C17—O2	108.22 (17)
C9—C8—H8A	111.1	O3—C17—H17A	110.1
C7—C8—H8A	111.1	O2—C17—H17A	110.1
C9—C8—H8B	111.1	O3—C17—H17B	110.1
C7—C8—H8B	111.1	O2—C17—H17B	110.1
H8A—C8—H8B	109.0	H17A—C17—H17B	108.4
C10—C9—C1	119.9 (2)		
			
O1—C1—C2—C3	−0.8 (4)	C1—C9—C10—C11	178.6 (2)
C9—C1—C2—C3	179.0 (2)	C8—C9—C10—C11	1.0 (4)
O1—C1—C2—C7	−178.2 (2)	C9—C10—C11—C12	−171.8 (2)
C9—C1—C2—C7	1.6 (2)	C9—C10—C11—C16	8.7 (4)
C7—C2—C3—C4	1.6 (3)	C16—C11—C12—C13	−0.7 (3)
C1—C2—C3—C4	−175.5 (2)	C10—C11—C12—C13	179.7 (2)
C2—C3—C4—C5	1.0 (3)	C11—C12—C13—C14	−0.5 (3)
C3—C4—C5—C6	−2.0 (4)	C12—C13—C14—O3	−178.3 (2)
C4—C5—C6—C7	0.3 (3)	C12—C13—C14—C15	1.4 (3)
C5—C6—C7—C2	2.2 (3)	C17—O3—C14—C13	−175.9 (2)
C5—C6—C7—C8	−178.5 (2)	C17—O3—C14—C15	4.3 (2)
C3—C2—C7—C6	−3.2 (3)	C13—C14—C15—C16	−1.1 (4)
C1—C2—C7—C6	174.43 (19)	O3—C14—C15—C16	178.7 (2)
C3—C2—C7—C8	177.4 (2)	C13—C14—C15—O2	178.5 (2)
C1—C2—C7—C8	−5.0 (2)	O3—C14—C15—O2	−1.7 (3)
C6—C7—C8—C9	−173.2 (2)	C17—O2—C15—C16	177.9 (2)
C2—C7—C8—C9	6.1 (2)	C17—O2—C15—C14	−1.7 (2)
O1—C1—C9—C10	3.9 (3)	C14—C15—C16—C11	−0.2 (3)
C2—C1—C9—C10	−175.9 (2)	O2—C15—C16—C11	−179.78 (19)
O1—C1—C9—C8	−177.9 (2)	C12—C11—C16—C15	1.1 (3)
C2—C1—C9—C8	2.3 (2)	C10—C11—C16—C15	−179.4 (2)
C7—C8—C9—C10	172.9 (2)	C14—O3—C17—O2	−5.3 (2)
C7—C8—C9—C1	−4.9 (2)	C15—O2—C17—O3	4.3 (2)

### Hydrogen-bond geometry (Å, º)

**Table:**

*D*—H···*A*	*D*—H	H···*A*	*D*···*A*	*D*—H···*A*
C5—H5···O1^i^	0.95	2.47	3.290 (3)	144
C17—H17*A*···O1^ii^	0.99	2.46	3.302 (3)	143

Symmetry codes: (i) *x*−1/2, *y*, −*z*+3/2; (ii) *x*−1/2, −*y*+1/2, −*z*+1.
